# Understanding the role of gut microfloral bifidobacterium in cancer and its potential therapeutic applications

**DOI:** 10.20517/mrr.2023.51

**Published:** 2023-10-31

**Authors:** Devanshi Sharma, Devanshi Gajjar, Sriram Seshadri

**Affiliations:** Institute of Science, Nirma University, 382481 Ahmedabad, Gujarat, India.

**Keywords:** *Bifidobacterium*, gut microbiota, homeostasis, chemotherapy, carcinogenesis

## Abstract

Gut microbiota research has gained a tremendous amount of attention from the scientific community because of its contribution to gut homeostasis, human health, and various pathophysiological conditions. The early colonizer of the human gut, i.e., bifidobacteria, has emerged as an efficient probiotic in various diseased conditions, including cancer. This review explores the pros and cons of *Bifidobacterium* in various malignancies and various therapeutic strategies. We have illustrated the controversial role of bifidobacteria participating in various malignancies as well as described the current knowledge regarding its use in anticancer therapies. Ultimately, this article also addresses the need for further extensive research in elucidating the mechanism of how bifidobacteria is involved and is indirectly affecting the tumor microenvironment. Exhaustive and large-scale research is also required to solve the controversial questions regarding the involvement of bifidobacteria in cancer research.

## INTRODUCTION

Bifidobacteria are Gram-positive anaerobes, which are rod-shaped polymorphic bacteria containing a high G + C content. *Bifidobacterium* can be predominantly found in the mouth, ileum, colon, vagina, cervix, and sometimes in dental caries^[1]^. They are among the early colonizers of the gastrointestinal tract in humans^[2]^. The strains of the *Bifidobacterium* genus have gained a lot of attention mainly because of their beneficial effects on the host by being the most efficient probiotic. The diversity of bifidobacteria keeps changing throughout life, and it also gets affected by various other factors, including the type of delivery, formula feeding, breastfeeding, *etc.*^[3,4]^. Bifidobacteria play a significant role in maintaining gut homeostasis via different mechanisms, such as immune system modulation, pathogen elimination, antimicrobial activities, metabolite production, and the regulation of the intestinal epithelial barrier. Its role in gut homeostasis is further discussed at length in this article.

The beneficial role of using bifidobacteria in anticancer therapy has been explored very extensively. Interaction of *Bifidobacterium* with cell cycle regulatory proteins causes inhibition of the proliferating cancer cells via successful activation of pro-caspases and up-regulation of the *Bax* proteins which are pro-apoptotic in nature^[5]^. The specifics pertaining to the beneficial role of *Bifidobacterium* resulting in the generation of anticancer properties are due to their ability to fabricate short-chain fatty acids (SCFAs) that impede the growth and maturation of cancer cells. These mass-produced SCFA, namely butyrate, propionate, and acetate, also aid in alleviating inflammation, a major risk factor in the development of cancer^[6]^. Further, *Bifidobacterium* strains have showcased antitumor properties by conferring an increase in CD8^+^ T cells along with an increase in the CD8^+^/T Reg cells ratio^[7,8]^.

The recent exploration of the role of bifidobacteria as an enhancer for checkpoint inhibitor immunotherapy has revealed an increased efficacy of the treatment^[9]^. When administrated orally, it has shown an improved ability to control tumors, similar to PD-L1-specific antibody therapy, and when used in combinational therapy, the tumor outgrowth was nearly eradicated. This was possibly due to dendritic cell accumulation in the tumor microenvironment. Thus, cancer immunotherapy can certainly be modulated by bifidobacteria^[10]^. The exploration of using *Bifidobacterium* as a therapeutic agent for cancer treatment holds a promising way ahead. Some studies indicated the efficacy and enhancement of cancer immunotherapy due to the potential of bifidobacteria in the generation of antitumor responses. Researchers are also investigating its role in targeted cancer therapy by using it as a drug delivery system^[11]^. The area of bifidobacteria and cancer research is still perplexing and evolving due to the varied nature of different bifidobacterial strains influencing cancer prevention and development. Extensive research on the potential use of these microorganisms as a drug delivery tool for cancer prevention and combination therapy is a promising way ahead^[12]^.

While the role of bifidobacteria in positive outcomes of anticancer therapy is very well understood, their controversial negative role in cancer raises a lot of questions. Their relative abundance and additional indirect effects are reported, from which we can hypothesize that dysbiosis in bifidobacteria present in the gut can be an alarm linked with different malignant tumors. It is further explained in this article.

The review discusses the studies elucidating the role of bifidobacteria in maintaining gut homeostasis, the effects of its altered diversity in several cancer types, and its potential use as a therapeutic and delivery agent for therapeutic substances against cancer. The relationship described between bifidobacteria strains and neoplastic growth does not indicate a causal relationship. We are only trying to highlight the dysbiotic population, its various effects, and its relevance to cancer.

## *BIFIDOBACTERIUM*: A STAR PLAYER OF GUT HOMEOSTASIS

**Figure 1:**
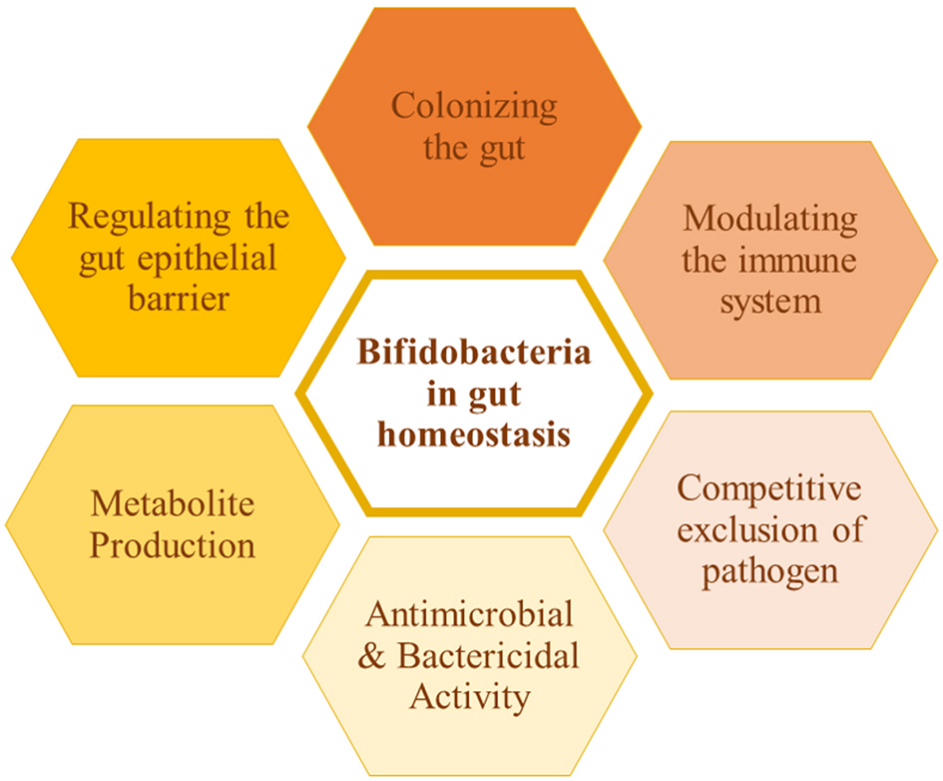
Role of bifidobacteria in gut homeostasis.

A growing body of evidence illustrates the role of bifidobacteria in maintaining human health by regulating intestinal homeostasis. The diversity analysis of bifidobacteria in various diseased conditions has been reported, in turn speculating its role in balancing the healthy gut microbiota composition. Any major shift in the gut microbial diversity can cause health problems. In order to balance the altered gut microbiota, various strategies including the use of probiotics, prebiotics, postbiotics, antibiotics, and fecal microbiota transplantation have been used. Bifidobacteria, recognized as a highly effective probiotic, offers numerous benefits to the host. According to the International Scientific Association for Probiotics and Prebiotics (ISAPP), probiotics can be defined as “live microorganisms that, when administered in adequate amounts, confer a health benefit on the host”^[13]^. Various mechanisms through which bifidobacteria exert benefits to the host include colonizing the gut, modulating the immune system, competitive exclusion of pathogens, antimicrobial and bactericidal activity, metabolite production, and regulating the gut epithelial barrier [Figure 1].

### Colonizing the gut

Colonization inside the gut is the primary and most significant mechanism of bifidobacteria in maintaining gut homeostasis. Bifidobacteria colonize the gut using various indigenous and exogenous factors. Prolonged adhesion to the gut epithelium is required for successful colonization, and several factors such as exopolysaccharides (EPS), lectins, adhesion molecules, and lipoproteins play an important role in this adhesion mechanism. These factors can help facilitate the binding of bifidobacteria to gut epithelium by working as “adhesion promoter”. These factors can be modified or exploited in such a way to increase the binding of the bifidobacteria to the intestinal epithelium. Increased expression of lipoprotein and BopA has been reported, which is significantly responsible for the adhesion of *B. bifidum* MIMBb75 to Caco-2 cells^[14]^. It has also been seen that human milk oligosaccharide and a probiotic contributed to the binding of *B. longum infantis* ATCC 15697 to Caco-2 cells and HT-29 cells^[15]^. Colonization by fimbrial attachment of certain strains like *B. longum* subsp. *longum* is also reported. Gene clusters responsible for pili synthesis have been identified in certain bifidobacterial strains like *B. breve* and *B. bifidum*. In *B. breve* UCC2003, a type IVb tight adherence (Tad) gene cluster has been identified, which enhanced the adhesion to the gut epithelium in murine models^[16]^. Exogenous factors like prebiotics can also enhance the adhesion of bifidobacteria inside the gut. Prebiotics can increase the survival rate of *Bifidobacterium* probiotics in the gut, which can, in turn, promote their growth^[17]^. Sometimes, exopolysaccharides also facilitate the adhesion of bifidobacteria to the gut epithelial wall. Studies have reported a correlation between the adhesion of bifidobacterial strains and EPS production. *B. breve A28* produces higher levels of EPS, which is responsible for its firm adhesion to the intestinal cell lines^[18]^.

### Modulating the immune system

Immunomodulatory effects of bifidobacteria exert various benefits to the host, and it is by far the most important mechanism by which bifidobacteria maintain gut homeostasis. Chiu *et al.* in 2014 published a study which showed that *B. adolescentis* DB-2458 and *B. longum* subsp. *infantis* GB-1496, which were isolated from human breast milk, shows a robust immunomodulatory effect via Th1/Th2-related cytokine regulation^[19]^. It has been seen that *B. breve* CNCM1-4035 and its culture supernatant can enhance the immune response of human intestinal dendritic cells via TLR signaling pathways against *Salmonella enterica typhi*. This can be attributed to the upregulation of TLR9 gene transcription, speculating that the TLR9 signaling pathway is responsible for robust anti-inflammatory effects against Salmonella^[20]^. *B. animalis* subsp. *lactis* BB-12 can reduce the proinflammatory cytokine secretion in adults by downregulating the TLR-2 expression^[21]^. In the case of cigarette smoking-induced immune response, *B. breve* M-16V and *L. rhamnosus* are known to suppress it when used in combination. In human THP-1 macrophage, a combination of *B. breve* M-16V and *L. rhamnosus* probiotics could suppress the expression of IL-1β, IL-6, IL-10, IL-23, TNF-α, and CXCL-8 along with TLR4, TLR9, and NF-κB signaling pathways which are mainly induced by cigarette smoking^[22]^. Apart from this, animal studies have shown that intestinal cells that were treated with *B. longum*, *B. infantis*, and *B. youth* can decrease the expression of TLR-2 and TLR-4. These three strains also enhanced the gut epithelial barrier functions^[23]^.

### Competitive exclusion of pathogens

Preventing the entry of pathogens by blocking their site of access is one of the mechanisms used by bifidobacteria to maintain gut health. Bifidobacteria can sometimes work in a competitive way, i.e., by binding to the sites of entry of enteric pathogens^[24,25]^. It has been seen that the growth of enteropathogenic and enterotoxigenic bacteria was inhibited by *B. breve* CNCM I-4035, and several other bifidobacterial species also negatively affected the binding of pathogens to the mucus layer of gut epithelium, ultimately preventing their entry^[26]^. Sometimes, bifidobacteria can also displace the pathogens that have already been colonized. For example, bifidobacteria can sequester the iron in the gut, which will deprive enteropathogenic bacteria of iron, thereby inhibiting their growth^[27]^.

### Antimicrobial and bactericidal activity

Studies have reported that fourteen strains of *Bifidobacterium*, which were isolated from the fecal samples of the infants, show antimicrobial activity against *S Typhimurium* SL1344. It can be because of the prevention of entry or intracellular inhibition of pathogens^[28]^. Bacteriocins isolated from *Bifidobacterium* called Bifidocins are also reported^[29]^. Bifidocin A, which was isolated from *B. animalis* BB04, can cause cell lysis of certain Gram-positive and negative bacteria and sometimes yeasts^[30]^. Bifidocin B, which was first isolated from *B. bifidum* NCFB 1454, has bactericidal activity against Gram-positive bacteria. It works by adhering to the cell surface receptors of the pathogen in a pH-dependent manner and initiating cell death^[31]^. *Bifidobacterium sp.* produces acidocin B that works against *Clostridium sp.* which can be found in fermented food items. Several other bacteriocins such as bifidin I from *B. bifidum*, thermophilicin from a thermophilic strain *B. thermophilum*, bifilact from *B. lactis*, and bisin from *B. longum* are also reported. These bacteriocins are stable in extreme temperatures and are also resistant to enzymatic and salts reaction^[32]^. Thermostable strains of bifidobacteria can inhibit the growth of the typical antibiotic-resistant strains of *Helicobacter pylori*^[32]^. It has also been seen that some *Bifidobacterium* strains contain low molecular weight compounds (LMWs), which can inhibit the growth of pathogens. This is how bifidobacteria prevent the growth of pathogens inside the host and maintain homeostasis^[33]^.

### Metabolite production

Metabolites produced by gut resident bacteria show inhibitory effects on the pathogens. Acetate produced by bifidobacteria can inhibit the growth of *E. coli O157*:*H7.* It can reduce the pH in the intestine, inhibiting the growth of pathogens^[34,35]^. Other than this, butyrate and propionate produced by bifidobacteria can work against colon carcinoma, exerting anti-carcinogenic activity and ultimately regulating gut homeostasis^[36,37]^. The anti-carcinogenic role of vitamin B12 and folates produced by bifidobacteria is also explored. Polysaturated fatty acids produced by probiotic bifidobacterial strains participate in gut homeostasis by exerting anti-carcinogenic, anti-diabetic, and anti-inflammatory properties^[37,38]^.

### Regulating the gut epithelial barrier

Human gut epithelium harbors the largest site for the interaction between the outer environment and the host milieu. It comprises the outermost layer of mucous followed by an internal layer of epithelial cells and innermost lamina propria. The layer of epithelial cells contains tight junctions, which are responsible for maintaining the integrity of the gut epithelium^[38]^. Bifidobacteria play a prominent role in maintaining gut integrity. Studies with human intestinal cell models Caco-2 have shown that upon administration of *Bifidobacterium*, downregulation in the expression of certain proinflammatory cytokines was seen, which enhanced the transepithelial electrical resistance. 10^8^ CFU dose of *Bifidobacterium* can increase the expression of several tight junction proteins, including claudins, ZO-1, and occluding. Bifidobacteria can also enhance the activity of tight junctions by targeting the TLR2 pathway^[39-41]^. In addition to this, they can prevent the prolonged adhesion of pathogens by secreting a thick layer of mucus that is constantly being replaced. Certain bifidobacteria species can also prevent the TNF-α mediated disruption of the epithelial barrier^[42]^.

## *BIFIDOBACTERIUM* AND CANCER: AN UNEXPLORED CONTROVERSY

As mentioned earlier, the gut microbiota of an individual keeps changing with age and it also gets affected by a variety of factors. However, any major change in the diversity of gut microbiota, known as “gut microflora dysbiosis”, may lead to major consequences in terms of developing various diseases. It has been reported that any chaotic shift in the abundance of bifidobacteria also results in developing atopic diseases, irritable bowel syndrome (IBS), inflammatory bowel disease (IBD), colorectal cancer (CRC), celiac diseases, and sometimes obesity^[43]^. Several studies have shown both the direct and indirect involvement of bifidobacteria in carcinogenesis. This controversial role of bifidobacteria is discussed below in this article.

### *Bifidobacterium* and gastric cancer

According to the Global Cancer Observatory (GLOBOCAN) data from the year 2020, gastric cancer occurrence ranks fifth and is the fourth most lethal malignancy worldwide^[44]^. According to Lauren’s classification, gastric cancer has two subtypes: diffuse and intestinal. The intestinal type of gastric cancer shows a better prognosis where malignant cells are arranged in a glandular fashion and also have adhesive properties. On the contrary, the diffuse type of gastric cancer shows a very poor prognosis, with tumor cells being scattered in the stomach as they have non-adhesive properties^[45]^. A research study reported in the year 2021 showed the gut microflora diversity in patients with gastric carcinoma with its subtypes, patients with gastrointestinal stromal tumors (GIST), and healthy controls in Finland^[46]^. It was seen that gastric adenocarcinoma patients had the lowest gut microflora diversity than GIST patients and healthy control. Upon the diversity analysis of diffuse and intestinal-type gastric adenocarcinoma compared with GIST and healthy control, they found that only the diffuse subtype had a significantly lower abundance of the bifidobacteriaceae family^[46]^. In mice models, it was previously reported that a lower abundance of gut resident *Bifidobacterium* is linked with more aggressive tumors^[47]^. Hence, we can postulate that lower Bifidobacterium levels are associated with more aggressive gastric malignancies.

The most common risk factor for gastric cancer is *Helicobacter pylori* infection. *H. pylori* was declared a class-I carcinogen by the International Agency for Research on Cancer (IARC) in the year 1994^[48]^. *H. pylori* infection is responsible for developing gastric diseases such as gastric cancer, gastric ulcer, and duodenal ulcer. However, patients with *H. pylori* infection can remain asymptomatic, while some patients develop severe aggressive forms of gastric diseases like gastric cancer or gastric ulcer. In February 2021, a similar intestinal microflora analysis study was published, which mainly focused on the *H. pylori* infectious virulence genes along with gut microflora diversity in the patients of Trivandrum, Southwestern India^[49]^. It was seen that in Trivandrum, the most dominant *H. pylori* strain was of the VacAs1i1m1CagA+ genotype. It is important to note that among the patients with *H. pylori* infection, only a few developed severe gastric abnormalities such as gastric ulcers and gastric cancer. This indicates that there should be some other factor that plays a vital role in developing severe gastric diseases. For further analysis, they compared the gut microflora diversity of *H. pylori*-infected patients with gastric cancer or gastric ulcer (CA/GU-Hp+) with relatively milder gastric diseases like non-ulcer dyspepsia or gastritis with and without *H. pylori* infection (NUD/GAS-Hp+ and NUD/GAS-Hp-) and gastroesophageal reflux disease without *H. pylori* infection (GERD-Hp-). Among those four groups, the CA/GU-Hp+ group had the lowest gut microflora diversity. The most important finding was that it had the significantly lowest abundance of the *Bifidobacterium* genus with strains including *B. adolescentis*, *B. longum*, and *B. bifidum*. Further whole-genome metagenomic sequencing analysis of CA/GU-Hp+ and NUD/GAS-Hp+ groups showed seven *Bifidobacterium* species, which were present in very inadequate amounts in the CA/GU-Hp+ group than the other. Those species included *B. adolescentis*, *B. bifidum*, *B. breve*, *B. longum*, *B. moukalabense*, *B. pseudocatenulatum*, and *B. reuteri*. This study indicates that *H. pylori* infection alone is not sufficient to develop severe gastric abnormalities such as gastric cancer and gastric ulcers. A lower abundance of gut resident *Bifidobacterium* genus with specific strains plays a significant role in developing severe gastric malignancy.

Bifidobacteria are considered the earliest colonizers of the human gastrointestinal tract. The diversity of *Bifidobacterium* decreases as individuals age. Numerous published studies have revealed the amount and strain of *Bifidobacterium* in the early stages of life, adulthood, and older age. The most abundant *Bifidobacterium* species seen in infants are *B. infantis*, *B. breve*, *B. longum*, and *B. bifidum*. During adulthood, the levels of *Bifidobacterium* decrease significantly. The most abundant bifidobacterial species found in adults are *B. adolescentis*, *B. catenulatum*, and *B. longum*. It has been observed that in later stages of life, with an increase in age, levels of *Bifidobacterium* further decrease. However, there are no profiles of old-age specific *Bifidobacterium* species because of a lack of particular understanding of an “elderly age”. It can be said that aging affects the *Bifidobacterium* population^[50]^. Age is also one of the most salient risk factors for gastric cancer, along with the stage of the cancer. The correlation between gastric cancer and age is reported. A study published in the year 2022 reveals that there was a significant decrease in the survival of gastric cancer patients as age increased. They included three different age groups in the study: groups I, II, and III, with ages of < 65 years, 60-74 years, and > 75 years, respectively. Groups II and III showed significantly lower survival, indicating a vital role of age in gastric cancer^[51]^. American Society of Clinical Oncology also describes age as one of the risk factors for gastric cancer. Gastric cancer mostly occurs in people with age > 55^[52]^. Now, as mentioned earlier, bifidobacterial abundance starts decreasing with age, and thus, we can say that there might be an association between bifidobacteria, age, and gastric cancer. A lower abundance of bifidobacteria in elderly individuals may act as a risk factor for gastric cancer.

Thus, from this data, we can observe a lower abundance of *Bifidobacterium* participating in gastric malignancies and developing more aggressive gastric tumors. Nonetheless, more extensive research including gut microflora profiling with an emphasis on bifidobacteria levels is required.

### *Bifidobacterium* and pancreatic cancer

Pancreatic cancer is the most lethal malignancy of all, and by the time it is diagnosed, it has already reached an advanced stage with metastasis, poor prognosis, and a more aggressive form^[53]^. A study published in 2022 reported the gut microflora profiles in pancreatic cancer patients^[54]^. In pancreatic cancer patients, apart from other bacteria, bifidobacteria was also seen in significantly lower amounts. It was also negatively correlated with the neutrophil-to-lymphocyte ratio (NLR). Previous studies have shown that NLR is associated with a poor prognosis in the case of pancreatic cancer^[55]^. Thus, a decrease in *Bifidobacterium* levels, which is correlated with the NLR, plays a role in pancreatic malignancy. Additionally, lower levels of *Bifidobacterium* will not be sufficient to inhibit tumor growth via the TNF-α-mediated pathway, and therefore, they can participate in pancreatic cancer progression^[56]^. Thus, we can postulate that a relatively lower abundance of *Bifidobacterium* may be involved in pancreatic cancer progression and poor prognosis.

### *Bifidobacterium* and colorectal cancer

The incidence of colorectal cancer ranks third and is the second most deadly cancer globally^[57]^. A few studies have addressed the profiling of fecal microflora in the case of colorectal cancer. A research paper published in 2018 revealed the changes in the gut microbiota population in gastrointestinal neoplasm. In the case of rectal cancer, there was a significantly lower abundance of the Bifidobacteriaceae family and *Bifidobacterium* genus^[58]^. One of the characteristic features of rectal cancer is elevated frequencies of aneuploidy along with Tp53 gene mutation. Folic acid plays an important role in regulating the genomic stability and thereby preventing the tumor growth. Folate deficiency can not only aggravate the genomic damage and increase the genomic instability in normal cells but also aggravate the colorectal cancer progression and make the normal colonic epithelial cells susceptible to malignant transformations^[59,60]^. Folate is not naturally synthesized in the human body. However, it is most significantly synthesized in the gut by *Bifidobacterium*^[61]^. Decreased levels of *Bifidobacterium* will not be able to synthesize enough folate, and thus, it will elevate the risk of aneuploidies related to rectal cancer, leading to severity in rectal cancer cases.

One of the risk factors for colon cancer is β-glucuronidase, which plays a primary role in carcinogenesis. β-glucuronidase hydrolyses the glucuronides, leading to formation of aglycone and glucuronic acid. It also transfers glucuronides to alcohols, phenols, and carboxylic acids, in turn participating in the detoxification process. However, increased activity of this enzyme leads to a reduction in glucuronides by forming the conjugates of glucuronic acid with toxins, hormones, drugs, *etc.* This leads to an increased production of carcinogens in the body, eventually elevating the risk of colon cancer^[62]^.

In 2014, Molan *et al*. described the effect of two prebiotics, i.e., First Leaf (FL) and Cassis Anthomix 30 (CAM30), on fecal *Bifidobacteria*, *Lactobacilli*, *Bacteroidetes spp.*, *Clostridium spp*. and β-glucuronidase. The consumption of FL and CAM30 caused increased growth of probiotics like Bifidobacteria and Lactobacilli while also reducing the number of harmful Bacteroidetes and Clostridia. The improved growth of bifidobacteria reduces the β-glucuronidase activity, ultimately preventing the occurrence of colon cancer^[63]^. It is important to note that a reduced abundance of *Bifidobacterium* is reported in several colorectal cancer cases, and these lower levels of bifidobacteria will be inadequate to regulate the β-glucuronidase activity and further fail to prevent the risk of carcinogenesis. Nevertheless, the mechanism behind how bifidobacteria controls the activity of β-glucuronidase remains an enigma.

### *Bifidobacterium* and ovarian cancer treatment failure

Epithelial ovarian cancer is a rare type of ovarian cancer as well as one of the lethal malignancies found in females^[64]^. In August 2021, a study was published emphasizing the gut microbiota population in patients receiving chemotherapy^[65]^. Patients with platinum resistance showed a reduced diversity of gut microbiota in comparison to healthy controls and had an overabundance of Coriobacteriaceae and *Bifidobacterium*. It was observed that these microorganisms are lactate-producing and an increase in the lactate-producing bacteria would also elevate lactate production. The Warburg effect is one of the important hallmarks of cancer. In simple terms, it is the utilization of glucose by cancer cells under aerobic conditions with an increased level of lactic acid fermentation and elevated secretion of lactate^[66]^. This increased lactate stimulates cell proliferation and suppresses the antitumor activity, in turn promoting cell angiogenesis and metastasis. The overproduction of lactate would impair the efficacy of chemotherapy by accelerating tumor angiogenesis, immune evasion, metastasis, and epithelial-mesenchymal transition (EMT). Therefore, we can safely assume that the dysbiotic population of *Bifidobacterium* is involved in the failure of chemotherapy against ovarian cancer.

**Figure 2:**
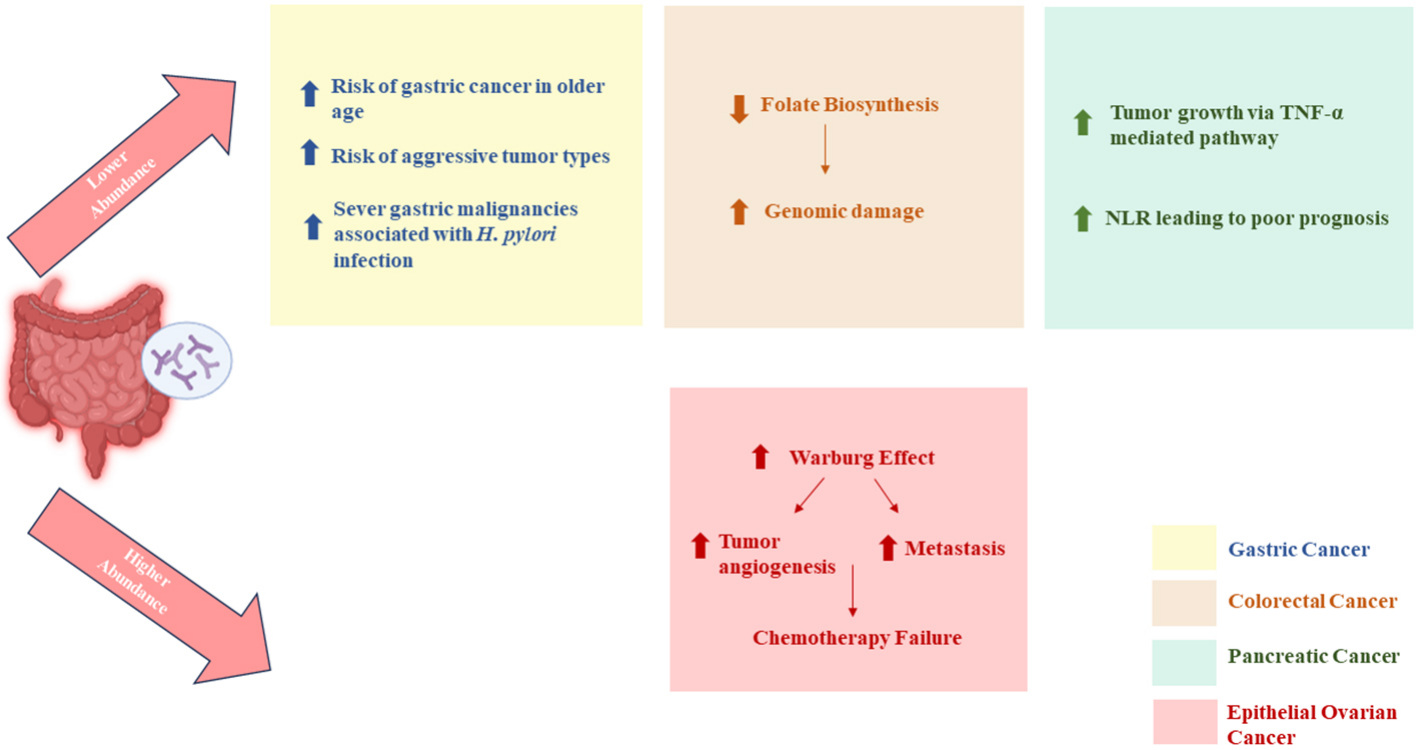
Diverse role of bifidobacteria in various cancer.

All these data regarding the role of bifidobacteria in various cancers shed light on its dysbiotic population in the gut. It is surely clear that there is insufficient substantial evidence regarding the gut bifidobacteria diversity in various types of cancers, but the already reported dysbiotic population should not be ignored. It indicates a potential danger towards the development of malignancies and their progression [Figure 2].

## *BIFIDOBACTERIUM* IN CONVENTIONAL CANCER THERAPY

Conventional cancer therapies broadly consist of surgical treatment and tumor removal by cauterization along with radiotherapy and/or chemotherapy for treating different kinds of tumors, such as hematological or solid tumors. The persistently evolving drug treatments for cancer and therapies have paved the way for the treatment of cancer via deeply innovative biotechnological, chemical, and biological drugs^[67]^.

Due to its ability to intensify the cancer cell apoptosis and shield against oxidative stress, the presence of *Bifidobacterium* in probiotics can potently induce anticancer effects. The combinative administration of probiotics along with anticancer drugs showed greater efficacy of antitumor activity in colorectal cancer. The common side effects caused by chemotherapy and radiation therapy, such as Chemotherapy-induced diarrhea (CID), mucositis, and Radiation-induced diarrhea (RID), were significantly reduced when probiotics containing *Bifidobacterium bifidum* were administered. Strains, namely *B. infantis*, *B. longum*, and *B. breve*, decreased proinflammatory cytokine levels when treated with 5-fluorouracil^[68]^. The clinical benefits of using *B. breve* probiotics were also seen in immunocompromised hosts during the chemotherapy by showing internal stabilization of intestinal microflora throughout this period^[69]^.

*B. infantis* impaired the severity of intestinal mucositis induced by chemotherapy^[70]^. Probiotics containing *Bifidobacterium* in combination with *Lactobacillus*, administrated as a 4-week post-surgical treatment for colorectal cancer, showed no aggravation for diarrhea, and they demonstrated immunobiotic effects by maintaining stable proinflammatory cytokine levels^[71]^. The oral administration of the probiotic bifidobacteria during the perioperative period of colorectal cancer resulted in a balanced ratio of intestinal microbiota and led to a lower inflammatory response, thereby promoting accelerated and healthy recovery post colorectal resection^[72]^. Cancer patients suffering from functional constipation were given *Bifidobacterium tetragenous viable bacteria* tablets along with chemotherapy, and the results indicated effective treatment of functional constipation^[73]^.

The pre-treatment of bifidobacterial strain *B. infantis* with its specific monoclonal antibody, when given in combination with radiation therapy, resulted in increased efficacy of radiation therapy for lung cancer. It particularly showed a delay in tumor growth, minimal toxicity, and prolonged survival in LLC xenograft mouse models^[74]^. Several research and clinical studies thus indicate that *Bifidobacterium* is vital in enhancing the efficacy of conventional cancer therapies such as surgery, chemotherapy, and radiation therapy when dispensed in combination with conventional therapies.

### Therapeutic potential of *Bifidobacterium*

The potential of bifidobacteria in prophylactic activities and therapeutic abilities have led to its popularity in the exploration of therapeutics. Bifidobacteria was identified as a potent resistant factor, which indicated that it is responsible for physiological homeostasis. However, when its population was imbalanced in a healthy person in ideal conditions, it showed no adversities. Interestingly, since every person is influenced by external forces, the dearth of this resistance factor can result in negative changes in the host’s body^[75]^.

Particular bacteria are being used as gene vectors due to qualities such as targeted delivery of genes and gene products to the specific tumor site and natural specificity for tumor. Bifidobacteria containing food-grade vectors resulted in an increased level of gene expression in systemic tumors over time. This promising result suggests the potential use of *B. longum* vectors for the potent delivery of therapeutic peptides at the tumor site^[76]^. This highlights the preferability of *Bifidobacterium* vectors for cancer gene therapy, as they are non-toxic and can proliferate in the tumor microenvironment, provided that the tumor maintains favorable hypoxic conditions^[77]^.

*Bifidobacterium* possesses multiple therapeutic abilities, such as anti-oxidant properties, pro-apoptotic activities, and anti-proliferative property that leads to anticancer actions. However, the exact mechanisms pertaining to the anticancer actions are not yet fully researched. Genetically modified *B. longum* is being investigated for its potent use in enzyme/prodrug therapies for the production of cytosine deaminase. *B. adolescentis* has recently been explored as a delivery system to deliver the antiangiogenic protein endostatin^[78]^. The use of bifidobacterial strains as supportive antitumor drugs still needs to be studied, especially in terms of clinical trials.

In the studies of treating breast cancers, a promising strategy was the use of *Bifidobacterium bifidum* to deliver PLGA, i.e., poly lactic-co-glycolic acid nanoparticles containing Perflourohexane (PFH). It showed enhancement in breast cancer treatment through the application of high-intensity focused ultrasound (HIFU) for ablation, exhibiting no side effects but increased survival time of mice^[79]^.

The addition of *bifidobacteria* to fermented dairy products intensified its value as a superior therapeutic food supplement by effectively neutralizing dietary carcinogens^[80]^. Moreover, exploring the ability of *bifidobacteria* to colonize the tumor sites, along with further investigating optimal dosing, potential toxicity, and strategies to enhance site-specific colonization, holds promise for improving the efficacy of cancer treatment^[81]^.

### *Bifidobacterium* in immunotherapy

Gut microbiota composition can highly influence the type and intensity of immune responses generated by our body during a variety of diseases, especially autoimmune diseases and cancer^[82]^.

*Bifidobacterium spp*. modified the intestinal microbiota when administered orally, resulting in an alteration of the immune response to PD-L1 blockade and a significant increase in intratumor and circulating CD8^+^ T cells which were tumor antigen-specific, thereby indicating a possible increase in surveillance by dendritic cells throughout the body. Increased abundance of *B. longum* in the stools of healing cancer patients showed increased infiltration of T-cells in the tumor microenvironment^[83]^. Research also suggests that using *Bifidobacterium* during the pembrolizumab treatment in lung cancer was beneficial by showcasing proficient antitumor activity and induced change in the population of CD45^+^ cells^[84]^.

Studies have also shown that Bifido strains *B. adolesentis*, *B. breve*, and *B. Longum* were involved in the enhancement of cancer immunotherapeutic drugs via oral administration. Bifidobacterial species have increased the anti-melanoma activity effects of induction of innate immunity. Moreover, a cocktail of bifidobacteria can potentially fight tumor growth and its further experimentation can be extended to other forms of cancer^[84]^. Strains of *B. adolescentis* not only stimulate the production of anti-inflammatory cytokines but also contribute to the decrease in the ulceration area and intestinal wall thickening. Furthermore, an *in-vivo* study has reported that the *Bifidobacterium bifidum* strain can potently induce the formation of regulatory Foxp3C T cells due to the presence of a cell surface β-glucan/galactan (CSGG), which showcases a powerful suppressive activity against experimental colitis. Certain components of *Bifidobacterium*, such as sortase-dependent pili, are involved in the activation of various signals in macrophages due to the induction of TNF-α locally while simultaneously reducing proinflammatory cytokine expressions. *B. bifidum* strain produces two extracellular molecules, namely BopA and TagA. BopA, as a surface-associated protein, can stimulate the production of IL-8 and increase bifidobacterial adhesion to epithelial cells. TagA acts like a peptidoglycan lytic enzyme, causing induction of the IL-2 and proliferation of dendritic cells^[85]^. *Bifidobacterium* also promotes local anti-CD47 immunotherapy due to their ability to accumulate within the tumor microenvironment and enhance the stimulation via STING signaling, i.e., stimulation of interferon genes^[86]^. *B. pseudolongum*-derived inosine showed modulation in checkpoint inhibitor therapy^[87]^. *Bifidobacterium* also enhanced the natural killer cells (NK cells) activation at proliferation at the tumor site via showcasing antitumor immunity.

**Figure 3:**
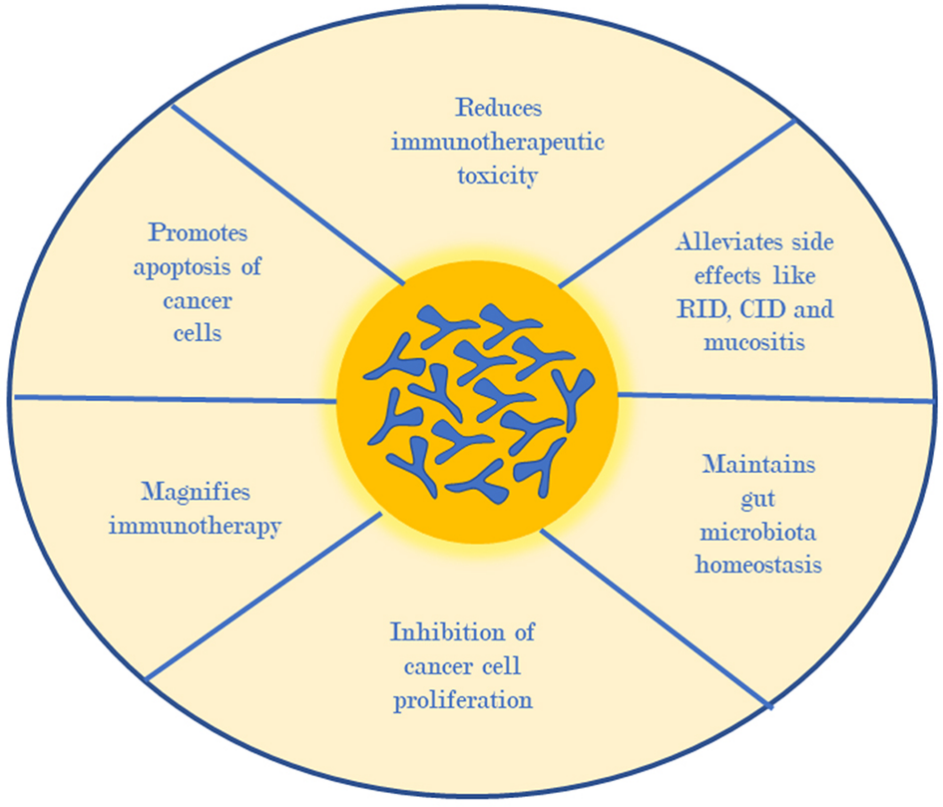
Role of bifidobacteria in cancer therapeutics.

Therefore, we can say that bifidobacteria are involved in cancer treatment by promoting the apoptosis of cancer cells, reducing immunotherapeutic toxicity, alleviating side effects such as RID, CID and mucositis, maintaining gut microflora homeostasis, inhibiting cancer cell proliferation, and magnifying the immunotherapy [Figure 3].

### *Bifidobacterium* and disease onset

It is certain that Bifidobacterium residing in the gut plays an important role in alleviating the symptoms of cancer. Moreover, the presence of Bifidobacterium in a higher ratio prevents harmful pathogens from getting attached to the intestinal wall and reduces the chances of occurrence of diseases such as IBS. Even though its beneficial role has been explored, further research still needs to be done to know how the Bifidobacterium plays a role in diseases apart from cancer.

Downregulation of *Bifidobacterium* levels has been reported not only in certain cancers but also in various other diseases. In the case of autoimmune hepatitis, a study published in 2020 showed a disease-specific decrease in the levels of *Bifidobacterium adolescentis*, *Bifidobacterium pseudocatenulatum*, and *Bifidobacterium longum*. These three species were associated with the severity of autoimmune hepatitis. Depletion of Bifidobacterium levels also resulted in failed remission^[88]^.

A study published in 2022 highlighted the association between the severity of SARS-CoV-2 infection and *Bifidobacterium* levels. Upon shotgun next-generation sequencing, it was found that the patients with SARS-CoV-2 infection had significantly depleted levels of *Bifidobacterium* and *Faecalibacterium*. Decreased abundance of *Bifidobacterium* was associated with increased severity of infection^[89]^.

A lower abundance of *Bifidobacterium* has also been seen in cases of wheezing diseases in Chinese children. Decreased abundance of bifidobacteria was seen in children with wheezing-stage asthma and children with bronchitis. This depletion in *Bifidobacterium* level was also negatively correlated with serum IgE levels, Th17-associated cytokine IL-17A, and Th2-associated cytokine IL-4. This study speculates the role of *Bifidobacterium* in altering the Th1/Th2/Th17 cell balance and, in turn, negatively affecting the prevention of wheezing diseases^[90]^.

Apart from respiratory tract infection, decreased bifidobacteria levels are also seen in metabolic diseases like type-1 and type-2 diabetes. In patients with long-standing type-1 diabetes, a significant decrease in *Bifidobacterium longum* was observed, which was also negatively correlated with myocardial infarction and macrovascular peripheral arterial disease^[91]^. Additionally, the patients with type-2 diabetes also had significantly lower levels of bifidobacteria compared to healthy controls^[92-94]^.

Liver diseases like non-alcoholic fatty liver disease associated hepatocellular carcinoma (NAFLD-HCC) are also reported to have depleted levels of bifidobacteria. NAFLD-HCC mice that were fed a high-fat, high-calorie (HFHC) diet have significantly decreased abundance of *Bifidobacterium pseudolongam*. In addition, oral dosing of *B. pseudolongum* resulted in the prevention of NAFLD-HCC^[95]^.

Analyzing the commensal interaction between the host and *Bifidobacterium*, with a particular emphasis on its impact on the intestinal lining, is crucial for comprehending its role. However, despite their increasing popularity, there is still a limited understanding of the impacts of probiotics on the indigenous elements of the human gut microbiota, as well as on the host organism^[96]^.

The presence of the infant-type *Bifidobacterium* plays a major role in modulating the immune system since infancy, with a possible indication that a reduced ratio of infant-type *Bifidobacterium* can result in a weaker immune system, potentially raising the risk of developing autoimmunity in adulthood^[97]^.

The production of neurotransmitters is a notable capability of bifidobacteria, as they are able to synthesize important neurotransmitters such as serotonin, gamma-aminobutyric acid (GABA), and acetylcholine. These neurotransmitters are essential for the regulation of mood, cognition, and behavior. When synthesized within the gastrointestinal tract, they can exert a direct influence on the central nervous system. It is imperative to acknowledge that investigations pertaining to the gut-brain axis are continuously advancing.

Although there is an increasing body of evidence supporting the impact of bifidobacteria on mental well-being, further research is required to fully understand the underlying mechanisms. Furthermore, it is important to note that there can be variations in individual responses to bifidobacteria, and the efficacy of a particular treatment may differ among individuals^[98]^.

There exists an association between reduced quantities of a certain gut microorganism, *Bifidobacterium*, and the presence of visceral obesity. Moreover, the association between *Bifidobacterium* abundance and visceral fat seems to be influenced by serum uric acid (SUA) levels. Moreover, A negative correlation between SUA levels and the abundance of *Bifidobacterium* is observed, suggesting that greater SUA levels were linked to lower levels of this particular bacterial species. In contrast, there was a positive correlation found between SUA and visceral fat area, indicating that elevated SUA levels were linked to greater amounts of visceral fat^[99]^.

Reduced levels of bifidobacteria have been observed in persons suffering from many medical disorders, such as infections, cystic fibrosis, hepatitis B, and both Type I and Type II diabetes mellitus. This trend suggests a possible role of bifidobacteria in the maintenance of overall physiological health. However, the exact cause-and-effect relationship between decreased levels of bifidobacteria and the development or advancement of these listed illnesses has yet to be definitively confirmed and clarified. Additional scientific investigation is necessary to determine the underlying mechanisms of this apparent correlation^[50]^.

Bifidobacteria play a major role in carbohydrate metabolism. Bifidobacteria do not employ the conventional Embden-Meyerhof-Parnas (EMP) route for the metabolism of hexose carbohydrates. In contrast, the researchers utilize a technique known as the “bifidshunt”, which specifically targets the enzyme fructose-6-phosphoketolase. The aforementioned pathway offers a notable benefit in terms of its ability to generate a greater amount of energy from carbs in comparison to the EMP pathway. In theory, the conversion of 1 mole of glucose yields 2.5 moles of ATP, along with 1.5 moles of acetate and 1 mole of lactate. The relative proportions of acetate and lactate can exhibit variability, contingent upon the specific carbohydrate substrate utilized and the developmental stage of the bacterial population. Moreover, the accelerated utilization of an energy substrate leads to heightened lactate production and diminished acetate production, whereas a slower utilization rate promotes elevated acetate production and reduced lactate production^[100]^. Thus, reduction in *Bifidobacterium* can be linked to metabolic dysfunction, thereby leading to metabolic disorders such as diabetes. Even though several studies have explored how the decreased ratio of bifidobacterial contributes to the onset of several diseases, further research still needs to be done with respect to its mechanisms.

Further studies on the molecular mechanisms involved due to the interaction between the host immune system and bifidobacteria can enhance the effectiveness of tumor-targeted immunotherapy.

Bifidobacteria, which are essential residents of the gastrointestinal tract, have a notable impact on overall well-being. Although the involvement of these bacteria in cancer has been recognized, additional investigation is required to fully appreciate their influence on different ailments. The presence of *Bifidobacterium* during infancy is of critical importance due to its role and regulation of early immune response; a decline in the bifidobacteria results in increased susceptibility to autoimmune disorders in later stages of life^[97]^. It also influences the CNS function and modulates mood and behavior by its capability to synthesize Ach and GABA^[98]^. However, further inquiry is necessary to achieve a thorough comprehension of their effects on mental well-being. Furthermore, there exists a correlation between diminished quantities of Bifidobacteria and the presence of visceral obesity^[99]^.

The dysbiotic population of *Bifidobacterium* has been seen in various diseases such as certain cancers, respiratory, hepatic, and metabolic diseases^[46,49,54,56,58,62,65,88,89,91,92,95]^. All these data indicate that alteration in bifidobacteria is involved in disease onset and progression. Establishing the dysbiotic level of bifidobacteria in different diseases would aid in using it as a therapeutic and prognostic marker. Nowadays, Bifidobacteria is also being used alongside chemotherapy against cancer. Bifidobacteria possessing immunobiotic, anti-proliferative, anti-oxidative, and pro-apoptotic properties help treat the cancer. Apart from that, it also participates in conserving an individual’s overall health by maintaining intestinal homeostasis. Therefore, more research regarding the use of *Bifidobacterium* with conventional therapies should be taken into consideration.

## CONCLUSION AND FUTURE PROSPECTIVES

Diversity, role, and function of gut microbiota have been extensively reported in connection to the host health. *Bifidobacterium*, one of the most important members of the commensal gut microbiota of the host, has been identified as a key player in cancer therapeutics. A lower abundance of certain strains of *Bifidobacterium* has been implicated in the progression of gastric, pancreatic, and colorectal cancer. *Bifidobacterium* also affects the Warburg effect, in turn playing a role in the failure of ovarian cancer treatment. It is important to note here that the cause-effect relationship between *Bifidobacterium* levels and cancer progression is difficult to assert. It is not clear whether the dysbiosis in bifidobacteria is exerting the tumor progressive activities or if other factors involved in the malignancies are causing the dysbiosis in the bifidobacteria population. From the already reported studies, we can only conjecture that the dysbiotic population of *Bifidobacterium* can be a potential threat to the development and progression of certain cancers.

Bifidobacteria has been explored for its anticancer potential. Bifidobacteria has been exploited as a delivery vector for the release of therapeutic agents with anti-inflammatory and tumor suppressor activities. Exploiting bifidobacteria as a cancer therapeutic alternative requires elaborate experimental evidence to evaluate its functional and mechanistic roles. This would help in understanding the potential of bifidobacteria as a “theragnostic marker”. Understanding the role of bifidobacteria individually and as a member of the gut microbiota consortium would open new arenas of exploration for cancer research.

## DECLARATIONS

### Copyright

© The Author(s) 2023.

## References

[1] Sgorbati B, Biavati B, Palenzona D

[2] Colston JM, Taniuchi M, Ahmed T (2022). Intestinal colonization with Bifidobacterium longum subspecies is associated with length at birth, exclusive breastfeeding, and decreased risk of enteric virus infections, but not with histo-blood group antigens, oral vaccine response or later growth in three birth cohorts. Front Pediatr.

[3] Dominguez-Bello MG, Costello EK, Contreras M (2010). Delivery mode shapes the acquisition and structure of the initial microbiota across multiple body habitats in newborns. Proc Natl Acad Sci U S A.

[4] Guaraldi F, Salvatori G (2012). Effect of breast and formula feeding on gut microbiota shaping in newborns. Front Cell Infect Microbiol.

[5] Nowak A, Paliwoda A, Błasiak J (2019). Anti-proliferative, pro-apoptotic and anti-oxidative activity of Lactobacillus and Bifidobacterium strains: a review of mechanisms and therapeutic perspectives. Crit Rev Food Sci Nutr.

[6] Coutzac C, Jouniaux JM, Paci A (2020). Systemic short chain fatty acids limit antitumor effect of CTLA-4 blockade in hosts with cancer. Nat Commun.

[7] Asadollahi P, Ghanavati R, Rohani M, Razavi S, Esghaei M, Talebi M (2020). Anti-cancer effects of Bifidobacterium species in colon cancer cells and a mouse model of carcinogenesis. PLoS One.

[8] Yoon Y, Kim G, Jeon BN, Fang S, Park H (2021). Bifidobacterium strain-specific enhances the efficacy of cancer therapeutics in tumor-bearing mice. Cancers.

[9] Wu J, Wang S, Zheng B, Qiu X, Wang H, Chen L (2021). Modulation of gut microbiota to enhance effect of checkpoint inhibitor immunotherapy. Front Immunol.

[10] Vétizou M, Pitt JM, Daillère R (2015). Anticancer immunotherapy by CTLA-4 blockade relies on the gut microbiota. Science.

[11] Li W, Zhang Z, Liu J (2022). Nanodrug-loaded Bifidobacterium bifidum conjugated with anti-death receptor antibody for tumor-targeted photodynamic and sonodynamic synergistic therapy. Acta Biomater.

[12] Benito I, Encío IJ, Milagro FI (2021). Microencapsulated *Bifidobacterium bifidum* and *Lactobacillus gasseri* in combination with quercetin inhibit colorectal cancer development in Apc^Min/+^ mice. Int J Mol Sci.

[13] Hill C, Guarner F, Reid G (2014). Expert consensus document. The international scientific association for probiotics and prebiotics consensus statement on the scope and appropriate use of the term probiotic. Nat Rev Gastroenterol Hepatol.

[14] Gleinser M, Grimm V, Zhurina D, Yuan J, Riedel CU (2012). Improved adhesive properties of recombinant bifidobacteria expressing the Bifidobacterium bifidum-specific lipoprotein BopA. Microb Cell Fact.

[15] Kavanaugh DW, O’Callaghan J, Buttó LF (2013). Exposure of Bifidobacterium longum subsp. infantis to milk oligosaccharides increases adhesion to epithelial cells and induces a substantial transcriptional response. PLoS One.

[16] Foroni E, Serafini F, Amidani D (2011). Genetic analysis and morphological identification of pilus-like structures in members of the genus Bifidobacterium. Microb Cell Fact.

[17] Sarkar A, Mandal S (2016). Bifidobacteria-insight into clinical outcomes and mechanisms of its probiotic action. Microbiol Res.

[18] Alp G, Aslim B (2010). Relationship between the resistance to bile salts and low pH with exopolysaccharide (EPS) production of Bifidobacterium spp. isolated from infants feces and breast milk. Anaerobe.

[19] Chiu Y, Tsai J, Lin S, Chotirosvakin C, Lin M (2014). Characterisation of bifidobacteria with immunomodulatory properties isolated from human breast milk. J Funct Foods.

[20] Bermudez-Brito M, Muñoz-Quezada S, Gomez-Llorente C (2013). Cell-free culture supernatant of Bifidobacterium breve CNCM I-4035 decreases pro-inflammatory cytokines in human dendritic cells challenged with Salmonella typhi through TLR activation. PLoS One.

[21] Meng H, Ba Z, Lee Y (2017). Consumption of Bifidobacterium animalis subsp. lactis BB-12 in yogurt reduced expression of TLR-2 on peripheral blood-derived monocytes and pro-inflammatory cytokine secretion in young adults. Eur J Nutr.

[22] Mortaz E, Adcock IM, Ricciardolo FL (2015). Anti-inflammatory effects of lactobacillus rahmnosus and Bifidobacterium breve on cigarette smoke activated human macrophages. PLoS One.

[23] Yang X, Gao XC, Liu J, Ren HY (2017). Effect of EPEC endotoxin and bifidobacteria on intestinal barrier function through modulation of toll-like receptor 2 and toll-like receptor 4 expression in intestinal epithelial cell-18. World J Gastroentero.

[24] Becerra JE, Coll-Marqués JM, Rodríguez-Díaz J, Monedero V, Yebra MJ (2015). Preparative scale purification of fucosyl-N-acetylglucosamine disaccharides and their evaluation as potential prebiotics and antiadhesins. Appl Microbiol Biotechnol.

[25] Chichlowski M, De Lartigue G, German JB, Raybould HE, Mills DA (2012). Bifidobacteria isolated from infants and cultured on human milk oligosaccharides affect intestinal epithelial function. J Pediatr Gastroenterol Nutr.

[26] Collado MC, Gueimonde M, Sanz Y, Salminen S (2006). Adhesion properties and competitive pathogen exclusion ability of bifidobacteria with acquired acid resistance. J Food Prot.

[27] Vazquez-Gutierrez P, Lacroix C, Jaeggi T, Zeder C, Zimmerman MB, Chassard C (2015). Bifidobacteria strains isolated from stools of iron deficient infants can efficiently sequester iron. BMC Microbiol.

[28] Liévin V, Peiffer I, Hudault S (2000). Bifidobacterium strains from resident infant human gastrointestinal microflora exert antimicrobial activity. Gut.

[29] Bali V, Panesar PS, Bera MB, Kennedy JF (2016). Bacteriocins: recent trends and potential applications. Crit Rev Food Sci Nutr.

[30] Liu G, Ren L, Song Z, Wang C, Sun B (2015). Purification and characteristics of bifidocin A, a novel bacteriocin produced by Bifidobacterium animals BB04 from centenarians’ intestine. Food Control.

[31] Klaenhammer TR (1993). Genetics of bacteriocins produced by lactic acid bacteria. FEMS Microbiol Rev.

[32] Collado MC, González A, González R, Hernández M, Ferrús MA, Sanz Y (2005). Antimicrobial peptides are among the antagonistic metabolites produced by Bifidobacterium against Helicobacter pylori. Int J Antimicrob Agents.

[33] (2023). Heravi F, Hu H. Bifidobacterium: host-microbiome interaction and mechanism of action in preventing common gut-microbiota-associated complications in preterm infants: a narrative review. Nutrients.

[34] Fukuda S, Toh H, Hase K (2011). Bifidobacteria can protect from enteropathogenic infection through production of acetate. Nature.

[35] Matsuki T, Pédron T, Regnault B, Mulet C, Hara T, Sansonetti PJ (2013). Epithelial cell proliferation arrest induced by lactate and acetate from Lactobacillus casei and Bifidobacterium breve. PLoS One.

[36] Bindels LB, Porporato P, Dewulf EM (2012). Gut microbiota-derived propionate reduces cancer cell proliferation in the liver. Br J Cancer.

[37] Ravishankar RV, Jamuna AB https://www.taylorfrancis.com/books/edit/10.1201/b17912/beneficial-microbes-fermented-functional-foods-ravishankar-rai-jamuna-bai.

[38] Vancamelbeke M, Vermeire S (2017). The intestinal barrier: a fundamental role in health and disease. Expert Rev Gastroenterol Hepatol.

[39] Hsieh CY, Osaka T, Moriyama E, Date Y, Kikuchi J, Tsuneda S (2015). Strengthening of the intestinal epithelial tight junction by Bifidobacterium bifidum. Physiol Rep.

[40] Ulluwishewa D, Anderson RC, McNabb WC, Moughan PJ, Wells JM, Roy NC (2011). Regulation of tight junction permeability by intestinal bacteria and dietary components. J Nutr.

[41] Ling X, Linglong P, Weixia D, Hong W (2016). Protective effects of Bifidobacterium on intestinal barrier function in LPS-induced enterocyte barrier injury of Caco-2 monolayers and in a rat NEC model. PLoS One.

[42] Al-Sadi R, Dharmaprakash V, Nighot P (2021). Bifidobacterium bifidum enhances the intestinal epithelial tight junction barrier and protects against intestinal inflammation by targeting the toll-like receptor-2 pathway in an NF-κB-independent manner. Int J Mol Sci.

[43] Tojo R, Suárez A, Clemente MG (2014). Intestinal microbiota in health and disease: role of bifidobacteria in gut homeostasis. World J Gastroenterol.

[44] Sung H, Ferlay J, Siegel RL (2021). Global cancer statistics 2020: GLOBOCAN estimates of incidence and mortality worldwide for 36 cancers in 185 countries. CA Cancer J Clin.

[45] Lauren P (1965). The two histological main types of gastric carcinoma: diffuse and so-called intestinal-type carcinoma. An attempt at a histo-clinical classification. Acta Pathol Microbiol Scand.

[46] Sarhadi V, Mathew B, Kokkola A (2021). Gut microbiota of patients with different subtypes of gastric cancer and gastrointestinal stromal tumors. Gut Pathog.

[47] Sivan A, Corrales L, Hubert N (2015). Commensal Bifidobacterium promotes antitumor immunity and facilitates anti-PD-L1 efficacy. Science.

[48] Feldman M, Friedman LS, Brandt LJ https://www.sciencedirect.com/book/9781416061892/sleisenger-and-fordtrans-gastrointestinal-and-liver-disease.

[49] Devi TB, Devadas K, George M (2021). Low Bifidobacterium abundance in the lower gut microbiota is associated with helicobacter pylori-related gastric ulcer and gastric cancer. Front Microbiol.

[50] Arboleya S, Watkins C, Stanton C, Ross RP (2016). Gut bifidobacteria populations in human health and aging. Front Microbiol.

[51] Choi Y, Kim N, Kim KW (2022). Gastric cancer in older patients: a retrospective study and literature review. Ann Geriatr Med Res.

[52] https://www.cancer.net/cancer-types/stomach-cancer/risk-factors.

[53] Hu JX, Zhao CF, Chen WB (2021). Pancreatic cancer: a review of epidemiology, trend, and risk factors. World J Gastroenterol.

[54] Wang XY, Sun ZX, Makale EC (2022). Gut microbial profile in patients with pancreatic cancer. Jundishapur J Microbiol.

[55] Szkandera J, Stotz M, Eisner F (2013). External validation of the derived neutrophil to lymphocyte ratio as a prognostic marker on a large cohort of pancreatic cancer patients. PLoS One.

[56] Kim Y, Lee D, Kim D (2008). Inhibition of proliferation in colon cancer cell lines and harmful enzyme activity of colon bacteria by Bifidobacterium adolescentis SPM0212. Arch Pharm Res.

[57] Deo SVS, Sharma J, Kumar S (2022). GLOBOCAN 2020 report on global cancer burden: challenges and opportunities for surgical oncologists. Ann Surg Oncol.

[58] Youssef O, Lahti L, Kokkola A (2018). Stool microbiota composition differs in patients with stomach, colon, and rectal neoplasms. Dig Dis Sci.

[59] Wang Z, Su C (2023). Effects of folic acid deficiency on genetic damage in colorectal cancer cells. Am J Transl Res.

[60] Kim Y (2016). Current status of folic acid supplementation on colorectal cancer prevention. Curr Pharmacol Rep.

[61] Sugahara H, Odamaki T, Hashikura N, Abe F, Xiao JZ (2015). Differences in folate production by bifidobacteria of different origins. Biosci Microbiota Food Health.

[62] Waszkiewicz N, Szajda SD, Konarzewska-Duchnowska E (2015). Serum β-glucuronidase as a potential colon cancer marker: a preliminary study. Postepy Hig Med Dosw.

[63] Molan AL, Liu Z, Plimmer G (2014). Evaluation of the effect of blackcurrant products on gut microbiota and on markers of risk for colon cancer in humans. Phytother Res.

[64] Webb PM, Jordan SJ (2017). Epidemiology of epithelial ovarian cancer. Best Pract Res Clin Obstet Gynaecol.

[65] D’Amico F, Perrone AM, Rampelli S (2021). Gut microbiota dynamics during chemotherapy in epithelial ovarian cancer patients are related to therapeutic outcome. Cancers.

[66] Warburg O (1956). On the origin of cancer cells. Science.

[67] Falzone L, Salomone S, Libra M (2018). Evolution of cancer pharmacological treatments at the turn of the third millennium. Front Pharmacol.

[68] Badgeley A, Anwar H, Modi K, Murphy P, Lakshmikuttyamma A (2021). Effect of probiotics and gut microbiota on anti-cancer drugs: mechanistic perspectives. Biochim Biophys Acta Rev Cancer.

[69] Wada M, Nagata S, Saito M (2010). Effects of the enteral administration of Bifidobacterium breve on patients undergoing chemotherapy for pediatric malignancies. Support Care Cancer.

[70] Mi H, Dong Y, Zhang B (2017). Bifidobacterium infantis ameliorates chemotherapy-induced intestinal mucositis via regulating T cell immunity in colorectal cancer rats. Cell Physiol Biochem.

[71] Zaharuddin L, Mokhtar NM, Muhammad Nawawi KN, Raja Ali RA (2019). A randomized double-blind placebo-controlled trial of probiotics in post-surgical colorectal cancer. BMC Gastroenterol.

[72] Mizuta M, Endo I, Yamamoto S (2016). Perioperative supplementation with bifidobacteria improves postoperative nutritional recovery, inflammatory response, and fecal microbiota in patients undergoing colorectal surgery: a prospective, randomized clinical trial. Biosci Microbiota Food Health.

[73] Liu J, Huang XE (2014). Efficacy of Bifidobacterium tetragenous viable bacteria tablets for cancer patients with functional constipation. Asian Pac J Cancer Prev.

[74] Yang J, Wu Z, Chen Y (2021). Pre-treatment with Bifidobacterium infantis and its specific antibodies enhance targeted radiosensitization in a murine model for lung cancer. J Cancer Res Clin Oncol.

[75] Arunachalam KD (1999). Role of Bifidobacteria in nutrition, medicine and technology. Nutrition Research.

[76] Cronin M, Morrissey D, Rajendran S (2010). Orally administered bifidobacteria as vehicles for delivery of agents to systemic tumors. Mol Ther.

[77] Yazawa K, Fujimori M, Amano J, Kano Y, Taniguchi S (2000). Bifidobacterium longum as a delivery system for cancer gene therapy: selective localization and growth in hypoxic tumors. Cancer Gene Ther.

[78] Michl P, Gress TM (2004). Bacteria and bacterial toxins as therapeutic agents for solid tumors. Curr Cancer Drug Targets.

[79] Tang Y, Chen C, Jiang B (2021). Bifidobacterium bifidum-mediated specific delivery of nanoparticles for tumor therapy. Int J Nanomedicine.

[80] Kailasapathy K, Chin J (2000). Survival and therapeutic potential of probiotic organisms with reference to Lactobacillus acidophilus and Bifidobacterium spp. Immunol Cell Biol.

[81] Ryan RM, Green J, Lewis CE (2006). Use of bacteria in anti-cancer therapies. Bioessays.

[82] Wu C, Wang X, Shang H, Wei H (2022). Construction of a humanized PBMC-PDX model to study the efficacy of a bacterial marker in lung cancer immunotherapy. Dis Markers.

[83] (2020). Chervin C, Gajewski TF. Microbiome-based interventions: therapeutic strategies in cancer immunotherapy. Immunooncol Technol.

[84] Rezasoltani S, Yadegar A, Asadzadeh Aghdaei H, Reza Zali M (2021). Modulatory effects of gut microbiome in cancer immunotherapy: a novel paradigm for blockade of immune checkpoint inhibitors. Cancer Med.

[85] Longhi G, van Sinderen D, Ventura M, Turroni F (2020). Microbiota and cancer: the emerging beneficial role of bifidobacteria in cancer immunotherapy. Front Microbiol.

[86] Shi Y, Zheng W, Yang K (2020). Intratumoral accumulation of gut microbiota facilitates CD47-based immunotherapy via STING signaling. J Exp Med.

[87] Kaźmierczak-Siedlecka K, Roviello G, Catalano M, Polom K (2021). Gut microbiota modulation in the context of immune-related aspects of Lactobacillus spp. and Bifidobacterium spp. in gastrointestinal cancers. Nutrients.

[88] Liwinski T, Casar C, Ruehlemann MC (2020). A disease-specific decline of the relative abundance of Bifidobacterium in patients with autoimmune hepatitis. Aliment Pharmacol Ther.

[89] Hazan S, Stollman N, Bozkurt HS (2022). Lost microbes of COVID-19: *Bifidobacterium*, *Faecalibacterium* depletion and decreased microbiome diversity associated with SARS-CoV-2 infection severity. BMJ Open Gastroenterol.

[90] Zhang L, Wan Y, Ma L, Xu K, Cheng B (2018). A low abundance of Bifidobacterium but not Lactobacillius in the feces of Chinese children with wheezing diseases. Medicine.

[91] van Heck JIP, Gacesa R, Stienstra R (2022). The gut microbiome composition is altered in long-standing type 1 diabetes and associates with glycemic control and disease-related complications. Diabetes Care.

[92] Gurung M, Li Z, You H (2020). Role of gut microbiota in type 2 diabetes pathophysiology. EBioMedicine.

[93] Sedighi M, Razavi S, Navab-Moghadam F (2017). Comparison of gut microbiota in adult patients with type 2 diabetes and healthy individuals. Microb Pathog.

[94] Wu X, Ma C, Han L (2010). Molecular characterisation of the faecal microbiota in patients with type II diabetes. Curr Microbiol.

[95] Song Q, Zhang X, Liu W

[96] Turroni F, Ribbera A, Foroni E, van Sinderen D, Ventura M (2008). Human gut microbiota and bifidobacteria: from composition to functionality. Antonie Van Leeuwenhoek.

[97] Lin C, Lin Y, Zhang H (2022). Intestinal 'infant-type' Bifidobacteria mediate immune system development in the first 1000 days of life. Nutrients.

[98] Alfonsetti M, Castelli V, d’Angelo M (2022). Are we what we eat? Impact of diet on the gut-brain axis in Parkinson’s disease. Nutrients.

[99] Gong H, Gao H, Ren Q, He J (2022). The abundance of bifidobacterium in relation to visceral obesity and serum uric acid. Sci Rep.

[100] O’Callaghan A, van Sinderen D (2016). Bifidobacteria and their role as members of the human gut microbiota. Front Microbiol.

